# Increased biomarkers of cardiovascular risk in HIV-1 viremic controllers and low persistent inflammation in elite controllers and art-suppressed individuals

**DOI:** 10.1038/s41598-022-10330-9

**Published:** 2022-04-21

**Authors:** Diogo Gama Caetano, Marcelo Ribeiro-Alves, Eugênio Damaceno Hottz, Larissa Melo Vilela, Sandra Wagner Cardoso, Brenda Hoagland, Beatriz Grinsztejn, Valdilea Gonçalves Veloso, Mariza Gonçalves Morgado, Patrícia Torres Bozza, Monick Lindenmeyer Guimarães, Fernanda Heloise Côrtes

**Affiliations:** 1 Laboratory of AIDS and Molecular Immunology, Oswaldo Cruz Institute – IOC, FIOCRUZ, Rio de Janeiro, RJ Brazil; 2 Laboratory of Clinical Research in STD and AIDS, National Institute of Infectology Evandro Chagas - INI, FIOCRUZ, Rio de Janeiro, RJ Brazil; 3 Laboratory of Immunothrombosis, Federal University of Juiz de Fora, Juiz de Fora, MG Brazil; 4 Laboratory of Immunopharmacology, Oswaldo Cruz Institute – IOC, FIOCRUZ, Rio de Janeiro, RJ Brazil

**Keywords:** Inflammation, Immunopathogenesis, Lymphocyte activation, Infection, Immunology, Adaptive immunity, Cardiovascular diseases, Platelets

## Abstract

HIV controllers (HICs) are models of HIV functional cure, although some studies have shown persistent inflammation and increased rates of atherosclerosis in HICs. Since immune activation/inflammation contributes to the pathogenesis of cardiovascular diseases (CVD), we evaluated clinical data and inflammation markers in HIV-1 viremic controllers (VC), elite controllers (EC), and control groups (HIV positive individuals with virological suppression by antiretroviral therapy—cART; HIV negative individuals—HIVneg) to assess whether they presented elevated levels of inflammation markers also associated with CVD. We observed the highest frequencies of activated CD8^+^ T cells in VCs, while EC and cART groups presented similar but slightly altered frequencies of this marker when compared to the HIVneg group. Regarding platelet activation, both HICs groups presented higher expression of P-selectin in platelets when compared to control groups. Monocyte subset analyses revealed lower frequencies of classical monocytes and increased frequencies of non-classical and intermediate monocytes among cART individuals and in EC when compared to HIV negative individuals, but none of the differences were significant. For VC, however, significant decreases in frequencies of classical monocytes and increases in the frequency of intermediate monocytes were observed in comparison to HIV negative individuals. The frequency of monocytes expressing tissue factor was similar among the groups on all subsets. In terms of plasma markers, VC had higher levels of many inflammatory markers, while EC had higher levels of VCAM-1 and ICAM-1 compared to control groups. Our data showed that VCs display increased levels of inflammation markers that have been associated with CVD risk. Meanwhile, ECs show signals of lower but persistent inflammation, comparable to the cART group, indicating the potential benefits of alternative therapies to decrease inflammation in this group.

## Introduction

HIV controllers (HICs) are HIV-infected individuals that present a spontaneous control of the viremia in the absence of antiretroviral treatment. These patients can be further classified as viremic controllers and elite controllers, based on the level of viremia control, and generally present a more preserved immunological setting^1^. Despite that, some HICs present higher levels of immune activation^2–6^ and inflammation^3,5,7–12^, which are hallmarks of HIV-1 infection associated with pathogenesis and disease progression^13^.

Besides the link with HIV progression, chronic immune activation and inflammation are also associated with an enhanced risk of non-AIDS-related diseases, such as metabolic syndrome and cardiovascular diseases (CVD) in people living with HIV-1 (PLHIV)^14,15^. These associations rely upon correlations between markers of immune activation, such as the frequency of CD38^+^HLA-DR^+^ T lymphocytes, with atherosclerosis and CVD among both the general^16^ and the HIV-1 infected population^17–20^. The same trend is observed for several inflammation markers, such as serum IL-6, IL-1β, C-reactive protein, and D-dimer levels, which are increased in HIV infection and associated with the development and severity of CVD^21–26^.

Several mechanisms link HIV infection, CVD, and inflammation. For example, inflammation is associated with endothelial activation, which leads to vasodilation, vascular permeability, and an increase in the expression of cellular adhesion molecules, such as VCAM-1 and ICAM-1, and chemokines, such as MCP-1^27^. These molecules are overexpressed in HIV infection and are essential for the pathogenesis of atherosclerosis^28–31^.

Different cells are affected by this process, but monocytes stand out due to their role in atherosclerosis^32–36^. In humans, these cells can be divided into three subsets, according to CD14 and CD16 expression. Most monocytes present the classical phenotype (CM; CD14^++^CD16^−^), characterized by a great response capacity against pathogens due to their high phagocytic potential and preferential differentiation to dendritic cells. In contrast, non-classical monocytes (NCM; CD14^+^CD16^++^) patrol endothelial and vascular tissues and are recruited to inflamed tissues, acting in tissue repair and inflammation control. Between both populations, an intermediary profile (IM; CD14^++^CD16^+^) is observed and associated with a pro-inflammatory response^37^. In the HIV infection, increased frequencies of CD16^+^ monocytes^38–45^ and monocyte activation markers, such as sCD14 and sCD163^3,7,18,23,40,41,46^, have been observed and correlated with disease progression and viral load. The same markers are also correlated with the development of CVD and the progression of atherosclerosis^3,18,24,32–36,44^.

Endothelial and monocyte activation are also associated with an increase in the expression of tissue factor (TF), a receptor and cofactor for (F)VII/VIIa, which activates coagulation cascade, correlates with atherogenesis, and is overexpressed during HIV infection^39,44,47–49^. Thus, TF can be a link between inflammation and the hyperactivation of platelets observed in HIV infection^30,50–55^, favoring peripheral arterial disease and ischemic events.

The persistence of inflammation and immune activation despite antiretroviral treatment (ART) resembles the profile observed for HICs in some studies, raising the question of whether these individuals are at a higher risk of CVD. In support of this, increased frequencies of CD16^+^ monocytes^4,9,45^, higher levels of TF^4^ and D-dimer^56^, and higher carotid intima-media thickness (cIMT)^3,12,57^ have been observed in some individuals, highlighting a possible impact on risk factors for the development of CVD. However, increased susceptibility to CVD in these individuals is still controversial, as some studies have shown an increased risk for EC^58^ and VC^59^, while others did not observe differences among HICs and individuals under ART^60–62^.

We investigated whether HICs could present an increased level of markers associated with CVD by evaluating several coagulation markers, immune activation, and inflammation in HICs with different levels of viremia control. Beyond T cell activation, we evaluated the frequency of activated platelets and the distribution of monocyte subsets, as well as their TF expression levels. We also measured the levels of markers of endothelial activation, mediators of the coagulation cascade, and cytokines involved in both inflammatory response and CVD development.

## Results

### Clinical and demographic characteristics of the cohort

We enrolled 13 HICs, classified as either elite controllers (n = 8) or viremic controllers (n = 5), and two age–sex-matched control groups composed of ART-suppressed HIV-1 infected individuals (cART; n = 18) and HIV-1-uninfected individuals (HIVneg; n = 18). Table 1 shows the clinical and demographic characteristics of the groups. No significant differences in age, sex, and race were observed among the groups. The median time since the HIV-1 diagnosis was 9, 14, and 17 years for EC, VC, and cART, respectively. The median time under HIV treatment was 11 years for the cART group. T CD4^+^ lymphocyte counts were similar among the groups, but VCs and cART had higher counts of T CD8^+^ lymphocytes. VCs had CD4/CD8 ratios below 1, while EC, cART, and HIVneg individuals had ratios above 1, with the last group presenting the higher ratios (*p* < 0.027). The median pVL for VCs was 700 copies/mL (QR 357-1480), while all EC and cART individuals had pVL < 40 copies/mL.

**Table 1:** Clinical and demographic characteristics of the study groups.

	HIVneg (n = 18)	cART (n = 18)	EC (n = 8)	VC (n = 5)	*p*-value
Age [median (IQR)]	48 (13)	52 (10)	44 (8)	46 (12)	0.225
Sex [%Male]	44.4%	61.1%	37.5%	60.0%	0.617
**Race**					
White [n (%)]	12 (66.7%)	9 (50%)	1 (12.5%)	1 (20%)	0.190
Black [n (%)]	2 (11.1%)	3 (16.7%)	3 (37.5%)	2 (40%)	
Mixed [n (%)]	4 (22.2%)	6 (33.3%)	4 (50%)	2 (40%)	
**Clinical Parameters of HIV-1 infection**					
Years since diagnosis [median of years (IQR)]	N/A	17 (11)	9 (3)	14 (8)	**0.014**
Years on ART [median of years (IQR)]	N/A	11 (9)	N/A	N/A	NA
Plasmatic viral load [copies/mL, median (IQR)]	N/A	< 40	< 40	700 (1123)	***p* < 0.001**
CD4 [cells/mm^3^, median (IQR)]	1069 (393)	1033 (457)	982 (186)	1043 (592)	0.918
CD8 [cells/mm^3^, median (IQR)]	571 (354)	967 (229)	616 (184)	1087 (256)	**0.003**
CD4/CD8 ratio [median (IQR)]	1.88 (0,76)	1.11 (0,16)	1.46 (0,69)	0.84 (0,56)	**0.027**
**Cardiovascular disease**					
Hypertension [n (%)]	3 (16.7%)	10 (55.6%)	2 (25%)	2 (40%)	0.092
Previous CVD [n (%)]	0 (0%)	6 (33.3%)	1 (12.5%)	0 (0%)	**0.027**
Family history [n (%)]	13 (72.2%)	14 (77.8%)	6 (75%)	5 (100%)	0.621
Framingham risk (BMI) [% (IQR)]	4.55% (7)	33% (27)	7% (5)	14.8% (10)	**0.037**
Framingham risk (Cholesterol) [% (IQR)]	3.8% (5)	16.7% (10)	4.4% (3)	8.7% (7)	0.076
**Other clinical events**					
Type II Diabetes [n (%)]	1 (5.6%)	6 (33.3%)	2 (25%)	2 (40%)	0.165
Dyslipidemia [n (%)]	1 (5.6%)	10 (55.6%)	1 (12.5%)	2 (40%)	**0.006**
Syphilis [Positive VDRL, n (%)]	0 (0%)	0 (0%)	0 (0%)	1 (20%)	0.329
**Life habits**					
Smoking [n (%)]	0 (0%)	2 (11.1%)	2 (25%)	1 (20%)	0.213
Physical activity [n (%)]	10 (55.6%)	7 (38.9%)	1 (12.5%)	1 (20%)	0.157
Previous use of cocaine [n (%)]	1 (5.6%)	3 (16.7%)	0 (0%)	1 (20%)	0.678
Alcohol associated risk					
Low	17 (94.4%)	17 (94.4%)	6 (75%)	3 (60%)	0.051
Moderate	1 (5.6%)	1 (5.6%)	2 (25%)	1 (20%)	
Very high	0 (0%)	0 (0%)	0 (0%)	1 (20%)	
**Anthropometry**					
BMI (median (IQR)	29 (5)	29 (6)	30 (5)	26 (3)	0.679
Systolic pressure [mmHg, (median (IQR)]	121 (12)	129 (27)	119 (18)	132 (31)	0.651
Diastolic pressure [mmHg, (median (IQR)]	75 (12)	77 (14)	72 (10)	94 (15)	0.183
Waist circumference [cm, (median (IQR)]	99 (17)	98 (21)	100 (5)	89 (15)	0.998
**Biochemistry**					
Glucose [mg/dl, median (IQR)]	91 (9)	95 (12)	96 (12)	105 (24)	0.077
Creatinine [mg/dl, median (IQR)]	0.83 (0,21)	0.90 (0,39)	0.77 (0,22)	1.07 (0,21)	0.267
**Complete blood count**					
Red blood cells [× 10^6^ cells/mm^3^, median (IQR)]	4.6 (0,5)	4.6 (0.6)	4.6 (1)	4.8 (0,3)	0.370
Monocytes [cells/mm^3^, median (IQR)]	476 (233)	482 (112)	383 (116)	343 (114)	0.059
Platelets [× 10^3^ cells/mm^3^, median (IQR)]	276 (84)	231 (62)	304 (65)	221 (47)	**0.025**
**Lipid profile**					
Total cholesterol [mg/dL, median (IQR)]	194 (37)	151 (66)	179 (30)	199 (76)	0.200
HDL [mg/dL, median (IQR)]	52 (21)	53 (25)	52 (15)	44 (14)	0.988
LDL [mg/dL, median (IQR)]	122 (25)	93 (58)	117 (30)	123 (37)	**0.044**
Triglycerides [mg/dL, median (IQR)]	76 (31)	95 (79)	83 (30)	165 (81)	0.061
Total cholesterol/HDL Ratio	4.6 (1,3)	4.3 (1,9)	4.3 (1,4)	4.4 (0.6)	0.325
LDL/HDL Ratio	3.1 (1,2)	2.6 (1,5)	3 (1,4)	2.6 (0,2)	0.199
VLDL [mg/dL, median (IQR)]	15 (6)	21 (17)	17 (6)	33 (16)	**0.022**

*HIVneg* HIV-1-uninfected individuals, *EC* Elite Controllers, *VC* Viremic controllers, *cART* ART-treated HIV-1 infected individuals, *N/A* Non-applicable, *IQR* Interquartile range, *NI* Non informed.

*p*-values were obtained through Kruskal Wallis test and *p*-values < 0.05 were considered significant and highlighted in bold characters.

Concerning risk factors associated with CVD, significant differences were observed only for dyslipidemia, with higher frequencies among cART and VC individuals (*p* < 0.006). Among anthropometric and biochemistry markers, no significant differences were observed among the groups. Despite that, VCs presented glucose levels close to or above the reference limits (> 100 mg/dL). Lipid profile analyses showed differences for low-density lipoprotein (LDL) cholesterol and very low-density lipoprotein (VLDL) among groups. While cART, HIVneg, and EC presented lipid profiles below the undesirable limits according to the reference values, VCs presented medians of total cholesterol close to the undesirable limit and medians of triglycerides and VLDL above reference limits (Table 1).

ART at the sample collection point for each cART individual is shown in Supplementary Table 1. The most prevalent cART regimens were the combination of two NRTIs plus one NNRTI (44%), followed by the combination of two NRTIs plus boosted PI (22%). Of those individuals using NRTI (89%), 81% were in use of TDF/3TC. Thirty three percent of the cART group used a boosted PI, and the most frequent regimens (66%) were DRV/RTV.

On cART and EC groups, some individuals presented previous CVD history. Among EC, a woman of advanced age was submitted for aortic valve replacement due to cardiac insufficiency 9 years before recruitment. Among cART individuals, two men presented acute myocardial infarction events, two men had a history of ischemic cardiopathy, a woman had aortic valve insufficiency, and another one presented with dilated myocardiopathy.

Further, all continuous measured variables analyzed in this study were pairwise comparisons among the studied groups and were performed by either the Mann–Whitney test or by T-tests, followed by Tukey’s honest significant difference control of family-wise type-1 error. Among the studied groups, the expected marginal mean values were estimated after multiple linear models including the confounders age, sex, and traditional risk factors for CVD (hypertension, dyslipidemia, diabetes, smoking, IMC, and total/LDL cholesterol).

### T cell activation status

**Figure 1:**
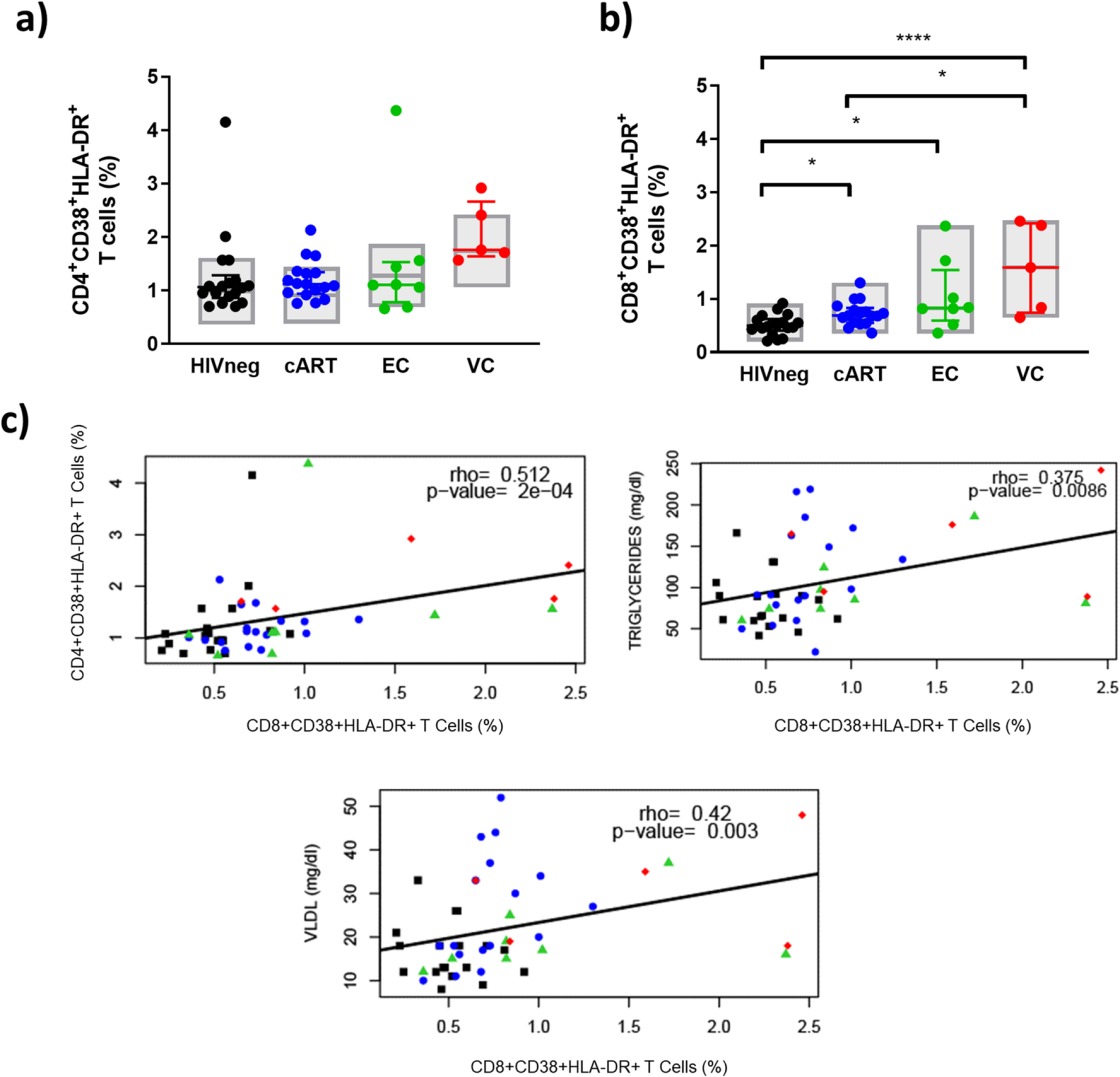
T cell activation levels among HICs and control groups. (**A**) Frequencies of CD4^+^ activated (CD38^+^HLA-DR^+^) T cells; (B) Frequencies of CD8^+^ activated (CD38^+^HLA-DR^+^) T cells; (C) Spearman correlations between T cell activation and other markers evaluated in the study; Colored horizontal bars represent the IQR and sample median, while gray boxplots represent linear model estimated marginal sample-bias adjusted means and its 95% confidence intervals (CI 95%). Comparisons of estimated marginal means among groups were performed by T-tests of contrasts obtained after multivariate-linear models fitted by ordinary least square regressions. *p*-values were corrected by the Tukey Honest Significant Difference post hoc method and represented as: *p < 0.05; **p < 0.01; ***p < 0.001; ****p < 0.0001. Comparison graphs were plotted with Graphpad Prism v9 and correlation graphs were plotted with R software v4.1.

First, we evaluated the frequency of CD4^+^ and CD8^+^ T cells HLA-DR^+^CD38^+^ as markers of the activation status of T lymphocytes.In the CD4^+^ subset, descriptive analyses indicated similar frequencies of CD38^+^HLA-DR^+^ cells between HIVneg, cART, and EC but increased frequencies in VC compared to HIVneg (*p* < 0.02). When adjusting for confounding factors, no significant differences were observed between the groups (Fig. 1a).

In the CD8^+^ subset, all HIV-positive groups had increased frequencies of CD38^+^HLA-DR^+^ cells compared to HIVneg individuals in a marginal sample-bias adjusted analysis (*p* < 0.04 for cART; *p* < 0.04 vs. EC; *p* < 0.0001 vs. VC) (Fig. 1b). Additionally, VC also presented increased frequencies of activated T cells when compared to cART (*p* < 0.03) and a tendency of increase in comparisons with EC (*p* = 0.055). Moreover, T CD8^+^ activation correlated positively and moderately with T CD4^+^ activation, and VLDL (Fig. 1c). A weak positive correlation was also observed between T CD8^+^ activation and triglycerides levels (Fig. 1c). These results indicate that although the highest levels of immune activation were observed for VC, a low level of CD8^+^ activation persists in EC and cART individuals, which was associated with metabolic and cardiovascular markers.

### Platelet activation status

**Figure 2:**
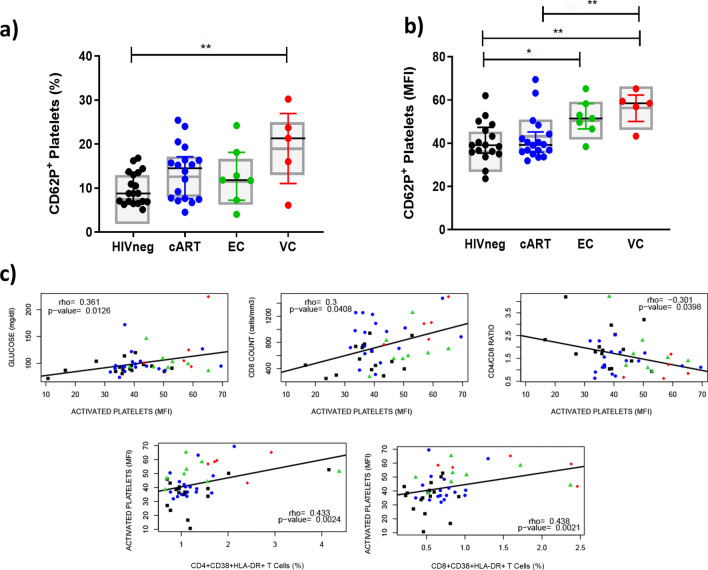
Frequency of activated platelets in HICs and control groups. (**A**) Frequencies of CD62P^+^ platelets; (**B**) Median fluorescence intensity of CD62P in the platelet population; (**C**) Spearman correlations between activation of platelets and other markers evaluated in the study; Colored horizontal bars represent the IQR and sample median, while gray boxplots represent linear model estimated marginal sample-bias adjusted means and its 95% confidence intervals (CI 95%). Comparisons of estimated marginal means among groups were performed by T-tests of contrasts obtained after multivariate-linear models fitted by ordinary least square regressions. *p*-values were corrected by the Tukey Honest Significant Difference post hoc method and represented as: *p < 0.05; **p < 0.01; ***p < 0.001; ****p < 0.0001. Comparison graphs were plotted with Graphpad Prism v9 and correlation graphs were plotted with R software v4.1.

To evaluate the existence of an altered state of coagulation related to HIV infection, we analyzed the frequencies of activated platelets in the HICs and control groups. We observed an increased frequency of CD62P^+^CD41^+^ platelets between VC and HIVneg, even after sample-bias adjustments (*p* < 0.01) (Fig. 2a). When the activation was analyzed in terms of the median fluorescence intensity (MFI) of CD62P in the CD41^+^ platelet population (Fig. 2b), higher values were observed in comparison to HIVneg, not only for VC (*p* < 0.007) but also for EC (*p* < 0.03), indicating increased activation in both HIC groups.

Moderate and positive correlations between CD62P MFI and both T CD8^+^ and CD4^+^ activation were verified (Fig. 2c). In addition, CD62P MFI also correlated weakly/positively with glucose levels and T CD8^+^ counts and weakly/negatively with the CD4/CD8 ratio (Fig. 2c).

These results indicate that both HIC groups, even ECs, present increased platelet activation in association with enhanced T cell responses.

### Monocyte subsets

Due to the participation of monocytes in inflammation and CVD development, we also characterized the population of monocytes in HICs, evaluating the frequency of the different subsets of monocytes classical (CM; CD14^++^CD16^−^), intermediate (IM; CD14^++^CD16^+^), and non-classical (NCM; CD14^+^CD16^++^) and the expression of TF in each subset. Due to the low level expression of TF in unstimulated monocytes observed in preliminary experiments, we assessed those parameters in both unstimulated and LPS-stimulated samples. For most samples, PMA stimulus was associated with increased expression of TF in all subsets, a slight increase in the frequency of classical monocytes, and a decrease in the frequency of intermediate and non-classical monocytes (Supplemental Fig. 1).

**Figure 3:**
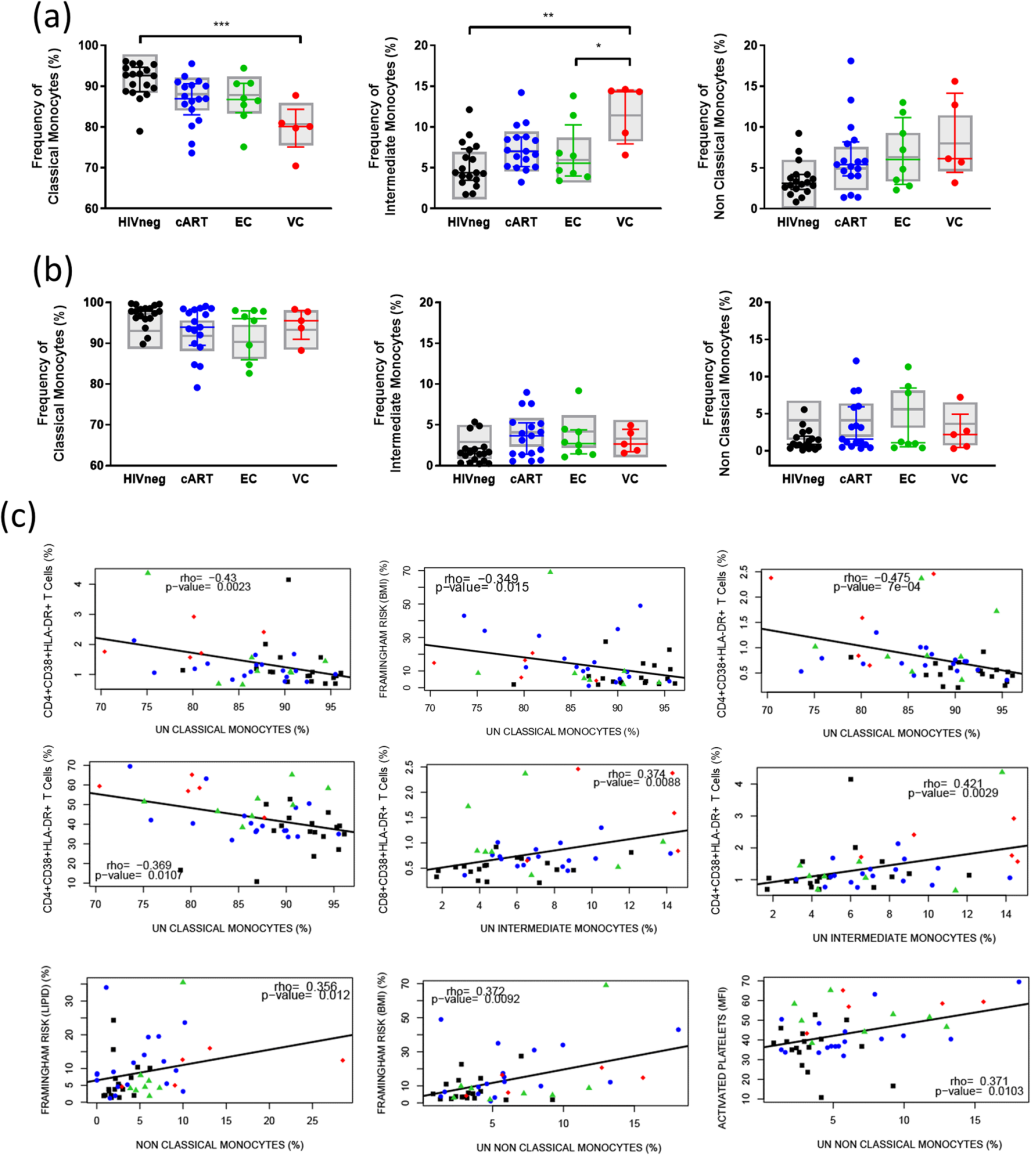
Frequency of monocyte subsets in HICs and control groups. (**A**) Frequencies of classical (CM; CD14^++^CD16^−^), Intermediate (IM; CD14^++^CD16^+)^ and non-classical monocytes (NCM; CD14^+^CD16^++^) in unstimulated samples; (**B**) Frequencies of CM, IM, and NCM in LPS-stimulated samples; (**C**) Significant Spearman correlations between frequency of unstimulated CM, IM and NCM and other markers evaluated in the study; Colored horizontal bars represent the IQR and sample median, while gray boxplots represent linear model estimated marginal sample-bias adjusted means and 95% confidence intervals (CI 95%). Comparisons of estimated marginal means among groups were performed by T-tests of contrasts obtained after multivariate-linear models fitted by ordinary least square regressions. *p*-values were corrected by the Tukey Honest Significant Difference post hoc method and represented as: *p < 0.05; **p < 0.01; ***p < 0.001; ****p < 0.0001. Comparison graphs were plotted with Graphpad Prism v9 and correlation graphs were plotted with R software v4.1.

Despite lower medians of CM and increased medians of IM and NCM for the three HIV-infected groups compared to HIVneg, statistical analyses revealed significant differences only for VC. For this group, decreased frequencies of CM (*p* < 0.001 vs. HIVneg) and increased frequency of IM (*p* < 0.002 HIVneg; *p* < 0.04 vs. EC) were observed (Fig. 3a). For LPS stimulated samples (Fig. 3b), no significant differences were observed, not even in descriptive analysis.

Spearman correlation analyses of unstimulated samples revealed moderate and negative correlations between CM and T cell activation in both CD4^+^ and CD8^+^ T cells subsets, plus weak and negative correlations between CM and BMI Framingham risk platelet activation (Fig. 3c). In contrast, weak and positive correlations were observed for NCM vs. BMI/lipid Framingham risk and platelet activation. Positive correlations were also observed IM versus T cell activation, although correlations with CD4^+^ T cells activation were moderate and with CD8^+^ T cells were weak (Fig. 3c).

**Figure 4:**
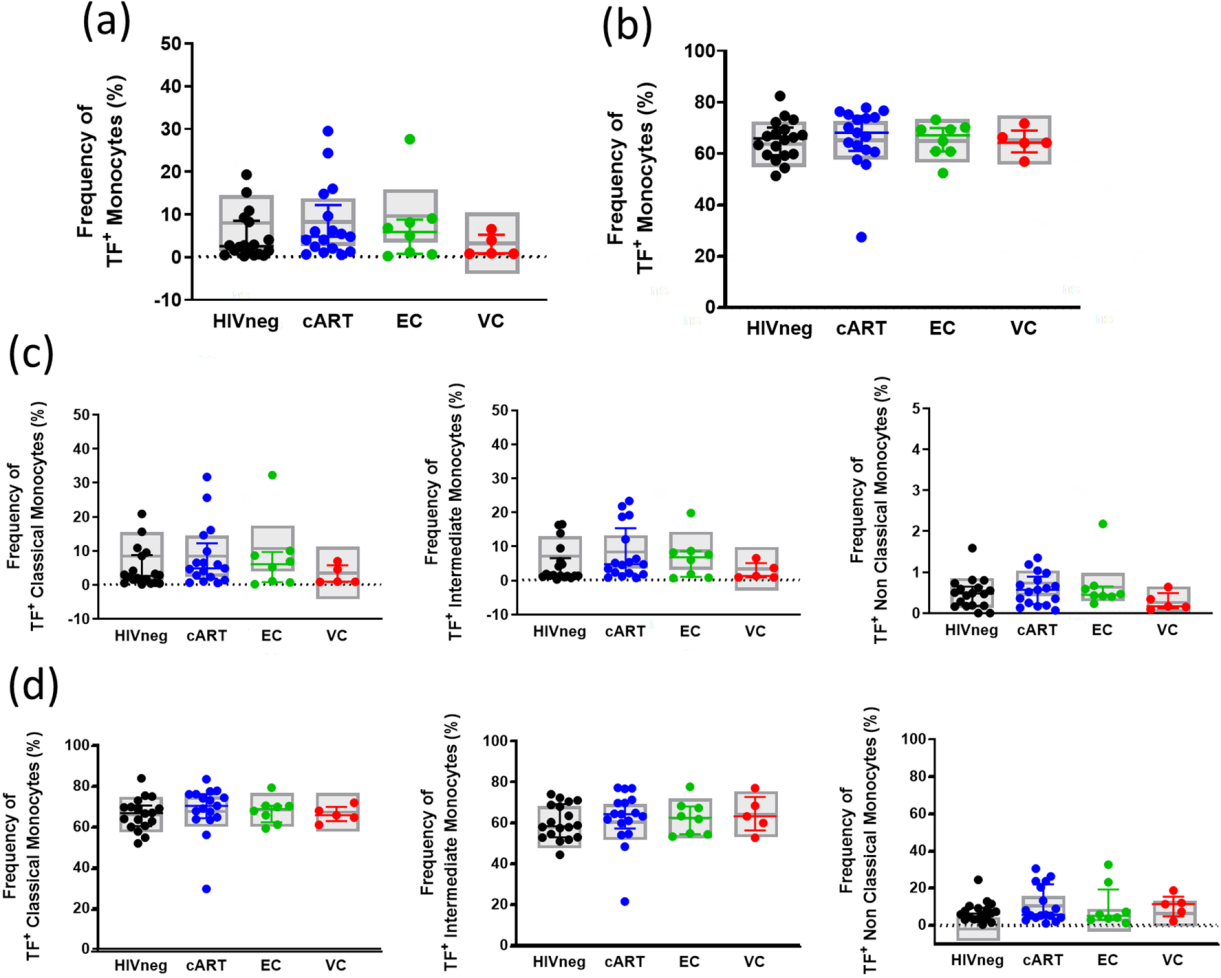
Frequency of TF^+^ monocytes in each subset in HICs and control groups. (**A**) Frequencies of TF^+^ monocytes in unstimulated samples; (**B**) Frequencies of TF^+^ monocytes in LPS-stimulated samples; (**C**) Frequencies of TF^+^ classical monocytes, intermediate and non-classical monocytes in unstimulated samples; (**D**) Frequencies of TF^+^ classical monocytes, intermediate and non-classical monocytes in LPS-stimulated samples; Colored horizontal bars represent the IQR and sample median, while gray boxplots represent linear model estimated marginal sample-bias adjusted means and its 95% confidence intervals (CI 95%). Comparisons of estimated marginal means among groups were performed by T-tests of contrasts obtained after multivariate-linear models fitted by ordinary least square regressions. *p*-values were corrected by the Tukey Honest Significant Difference post hoc method and represented as: *p < 0.05; **p < 0.01; ***p < 0.001; ****p < 0.0001. Comparison graphs were plotted with Graphpad Prism v9.

Finally, TF expression analyses did not show any differences among the groups for any evaluated subset (Fig. 4), nor for the stimulated or unstimulated samples, indicating similar levels of monocyte procoagulant activation in HICs and control groups. Concerning the previous results, monocyte subset analyses also showed that VC presents a background consistent with increased inflammation. EC and cART data indicate that a low level of inflammation exists, but it could be related to confounding factors.

### Plasma inflammatory markers

**Figure 5:**
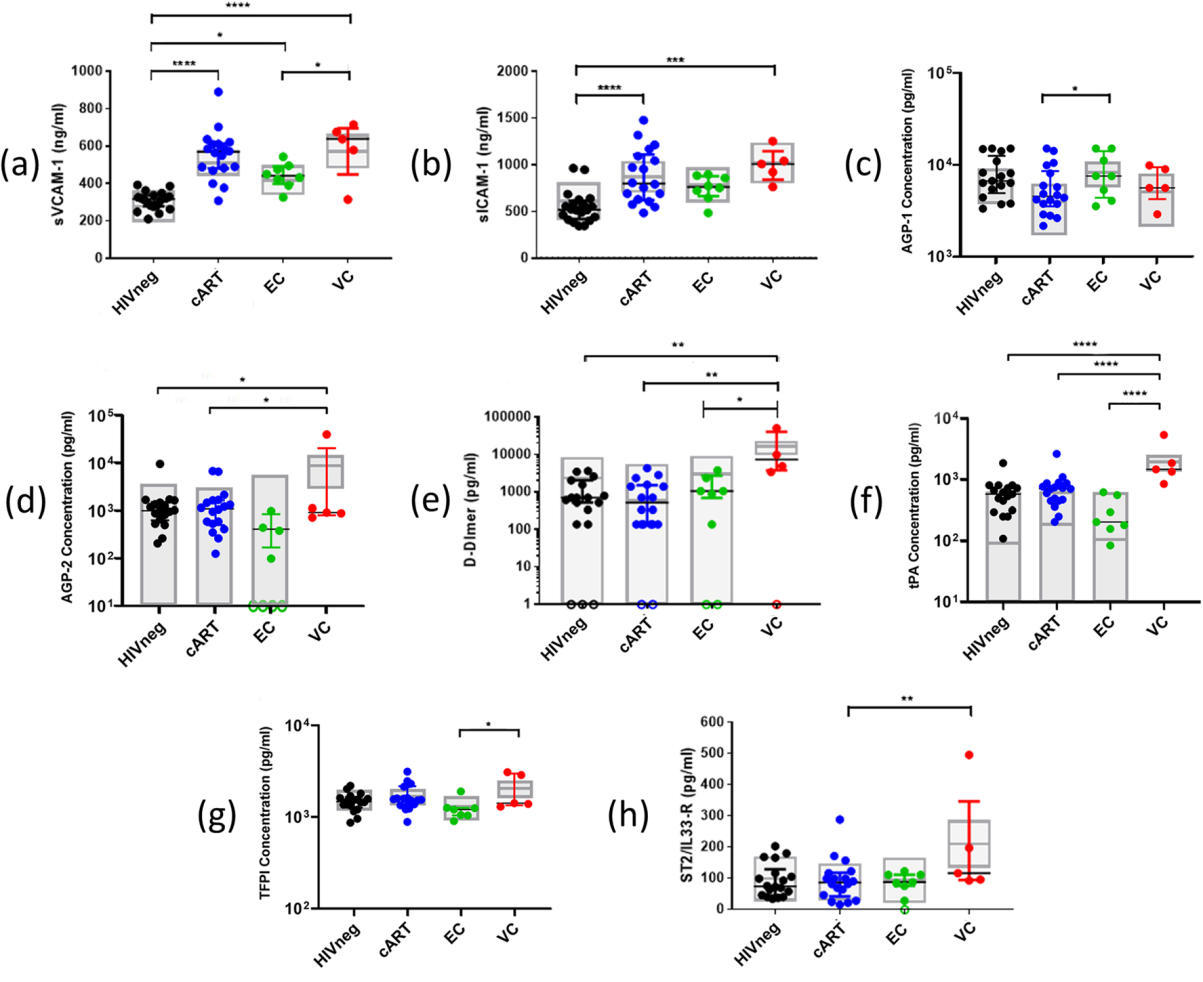
Plasma levels of inflammatory markers in HICs and control groups. The graphs represent the concentrations measured by multiplex Luminex or ELISA assay of: (**a**) sVCAM-1; (**b**) sICAM-1; (**c**) AGP-1; (**d**) AGP-2; (**e**) D-dimer; (**f**) tPA; (**g**) TFPI; (**h**) ST2/IL-33R; Open circles on the X axis represent samples with undetectable levels of the marker; Colored horizontal bars represent the IQR and sample median, while gray boxplots represent linear model estimated adjusted means and 95% confidence intervals (CI 95%). Comparisons of means among groups were performed by contrasts/differences obtained after both bi- and multivariate-linear models fitted by ordinary least square regressions. *p*-values were corrected by the Tukey Honest Significant Difference post hoc method and represented as: *p < 0.05; **p < 0.01; ***p < 0.001; ****p < 0.0001. Comparison graphs were plotted with Graphpad Prism v9.

In addition to cellular subsets related to inflammation/coagulation, we also measured the plasmatic levels of biomarkers commonly associated with the inflammatory response, endothelial activation, coagulation cascade, and/or pathogenic processes involved in the development of CVD (Fig. 5 and Supplementary Fig. 2).

Among the markers related to endothelial activation, we observed increased levels of VCAM-1 in the groups of HIV-positive individuals when compared to HIVneg after adjustment for confounding factors (*p* < 0.00001 for cART; *p* < 0.02 for EC; *p* < 0.0001 for VC). For ICAM-1, a significant increase compared to HIVneg was also observed for cART and VC groups (*p* < 0.05; *p* < 0.02, respectively). In addition, VCs presented increased levels of AGP-2 compared to cART and HIVneg (*p* < 0.02 for both), and cART presented decreased levels of AGP-1 compared to EC (*p* < 0.03) only after the sample-bias adjustment and marginal mean estimations. For MCP-1 and CD40L, no significant differences were observed between the groups, although most EC subjects presented undetectable levels of MCP-1 (Supplementary Fig. 2).

For soluble markers related to coagulation cascade, VCs presented higher levels of D-dimer (*p* < 0.01 vs. HIVneg; *p* < 0.002 vs. cART; *p* < 0.03 vs. EC) and tPA (*p* < 0.0001 vs. all) in comparison to all other groups. For EC, the descriptive analyses also showed lower levels of tPA (vs. cART; *p* < 0.05), TFPI (vs. VC; *p* < 0.04), and of vWF-A2 (vs. cART and VC; p < 0.005 and *p* < 0.03, respectively), but the difference did not remain significant after sample-bias adjustment and marginal mean estimations. Lastly, PAI-1 and sCD62P levels did not differ between the groups, while sTF levels were mostly undetectable for all samples (Supplementary Fig. 2).

In addition, we also measured levels of ST2, a biomarker of cardiac stress, and cytokines classically related to inflammation. For ST2, VCs presented higher levels than cART (*p* < 0.03). For IL-6, IL-10, and TNF-α, most samples had undetectable levels in the Luminex assay (Supplementary Fig. 2).

### Correlations between clinical, cellular, and plasmatic markers

Pearson correlations with *p*-value < 0.05 for all evaluated markers are shown in Supplementary Figs. 3 and 4. Among markers related to T cell activation (Supplementary Fig. 3), CD8 counts correlated positively and weakly with TFPI and vWF-A2, but moderately with ICAM, and VCAM. ICAM also correlated weakly/negatively with CD4/CD8 ratio. For activated T cell frequencies, T CD8^+^ activation correlated moderately/positively with VCAM and weakly/negatively with PAI-1 levels, highlighting the protective feature of the last.

For coagulation markers evaluated in this study (Supplementary Fig. 4a, the frequency of activated platelets correlated moderately/positively with MCP-1 concentrations, while CD62P MFI on platelets correlated weakly/negatively with PAI-1. In addition, moderate and positive correlations were observed for the TFPI/tPA and vWF-A2/tPA pairs.

Correlations were also observed between serum markers and monocyte subsets (Supplementary Fig. 4b). Concentrations of vWF-A2 correlated weakly, while ICAM and VCAM correlated moderately with the frequency of CM (all negative correlations). Instead, positive and moderate correlations were observed between the frequency of IM with vWF-A2 and VCAM. For NCM, moderate and positive correlations were observed with both VCAM and ICAM.

Finally, moderate and positive correlations among the endothelial activation markers included VCAM with ICAM and CD40L with AGP-1 (Supplementary Fig. 4c).

## Discussion

The association between chronic inflammation and immune activation with susceptibility to CVD is well established in PLHIV^14,15,22^. Nevertheless, inconsistent or contradictory reports about the persistence of immune disbalance in HICs, especially in EC, raise the question about the risk of developing HIV-associated comorbidities in these individuals. To clarify this, we evaluated biomarkers related to inflammation, immune activation, and CVD pathogenesis that could denote an increased risk for non-AIDS-related events in HICs despite the viremia control.

Beyond identifying CVD risk in HICs, the characterization of immunological alterations in these individuals compared to ART-suppressed individuals is relevant for the debate about the benefit of ART to HICs. In recent years, studies of ART effect in HICs showed slight improvements for CD4^+^ T cell counts^63,64^, HIV DNA/RNA levels^65–67^, and immune cellular activation^65,67^, but no impact on inflammation^56,65^. In addition, few studies evaluating the impact of ART among EC discriminate between persistent and transient viremia, although others showed markers that can distinguish both profiles^68,69^, and we do not know if ART affects these two subsets of EC differently, since ART effect in transient EC were only evaluated after the loss of virological control^68,70^. In light of this, and considering ART side-effects and challenges for adherence, more details about the immunological setting are needed to evaluate the cost–benefit of ART in HICs.

Since inflammation can be triggered by several factors and the risk of developing comorbidities is associated with factors such as age and sex, we adopted some strategies to reduce the impact of sample bias in the statistical analyses. First, we recruited matched control groups by age and sex to minimize the effect of these factors during analyses. Significantly higher frequencies of dyslipidemia were observed for the VC and cART groups, although all individuals with this profile were under statin treatment and had controlled cholesterol levels. In addition, we included confounding variables (e.g., age, sex, smoking, BMI, hypertension, dyslipidemia, diabetes, and total/LDL cholesterol levels) as they could be confounding factors in bivariate analyses and selected those correlated with outcomes (*p* < 0.02) for inclusion in the multivariate analysis.

Although essential for the immunological response, the immune activation observed in HIV infection is harmful in the long term, associated with AIDS progression^71–75^, and also CVD development^17–20^. In our study, the frequencies of activated CD8^+^ T cells differed for all HIV-infected groups compared to HIVneg. Although the difference between cART and HIVneg observed for this marker was minimal, our data corroborated that ART alone is not capable of normalizing levels of immune activation^76–79^ and that mechanisms other than viral replication may account for this persistence. Among those mechanisms, microbial translocation related to intestinal mucosa damage^8,80^, residual HIV replication due to low permeability of ART in anatomical sites^81–83^, and bystander activation of lymphocytes by pro-inflammatory cytokines^84,85^ are described.

These mechanisms should also be fueling the low level of activation observed for ECs. Although the results in this study contrast with previous results from our group that observed similar levels of activation between EC and HIVneg^6,79^, they agree with other studies that observed residual immune activation in EC^2–4^. In all cases, the immune activation observed for ECs was similar or lower to that observed in cART individuals, favoring the hypothesis that this state is driven by factors other than viral replication and that ART could not significantly impact these factors. On the contrary, VCs present higher activation than EC individuals, suggesting that VC could benefit from ART.

Apart from T cell activation, chronic inflammation is another element associated with immune activation during HIV infection that could be translated into several conditions, including the induction of a hypercoagulation disorder directly associated with the increased risk for thrombotic events in PLHIV^14,15,86,87^. In our study, we observed increased levels of some players related to coagulation activation in VC, such as the frequency of activated platelets and serum markers such as D-dimer, tPA, and vWF-A2, agreeing to previous studies that showed a hyperactivation state in platelets of PLHIV ^30,52,54,88^ and a correlation between some of these markers and the pVL^50,52^. Moreover, increased platelet activation and D-dimer, together with increased tPA levels, indicate that coagulation activation is compensated by increased fibrinolysis.

Increased activation of platelets was verified in both HIC groups compared to control subjects. To our knowledge, this is the first report indicating that HICs, including ECs, present an increased activation in platelets, which can contribute to ischemic and thrombotic events despite viremia control. No other differences were observed between cART and HIVneg groups, in contrast to other studies that observed the persistence of platelet activation despite ART use^53,54^. However, it is important to point out that in both studies, the individuals from the uninfected control group were younger than cART individuals, and this could have impacted the observed results, underlining the importance of strategies to minimize sample bias.

An imbalance among the different monocyte subsets in HIV-1 infection has been related to inflammation. In this context, our comparisons between the VC group and HIVneg agree with previous studies, which verified an increment in intermediary and non-classical populations and a decrease of CM in PLHIV^38–44^. Those observations point towards the role of viral replication in the induction of altered monocyte profiles, as seen in studies that correlated CD16^+^ monocytes, pVL, and disease progression^40,41,43^. However, our data indicate that other elements contribute to this imbalance rather than HIV infection in our groups of PLHIV, as similar trends of alterations were observed for EC and cART in unadjusted data, but not after adjustment for confounding factors.

The cART and EC groups presented altered medians for the three monocytes subsets compared to uninfected individuals; however, the differences were non-significant after statistical correction for multiple comparisons in the descriptive analysis after sample-bias adjustments. While the altered medians in ART-suppressed individuals resemble studies showing that ART is capable of improving but cannot normalize the frequencies of the different monocyte subsets^39,41,42,89^, for ECs, it relates to some level of alteration in monocyte dynamics, as previously observed in HICs studies^4,9,45^. However, the lack of significance for most comparisons and similarity to the cART group indicates that a residual imbalance in monocyte populations should be lower or similar to that observed for ART-suppressed individuals. In sum, the data obtained here for T cell and platelet activation status point towards the hypothesis that ART could not confer substantial clinical benefit to EC with high CD4^+^ T cell counts and low levels of inflammation^70^.

In addition to the monocyte subset frequencies, we did not observe differences among TF expression in these cells, even after stimuli with LPS, while plasma levels of this marker were mainly undetectable. Although TF is mainly produced by perivascular cells^90^, monocytes can also express this molecule in inflammatory settings or in response to LPS stimuli^48,91^. Thus, TF is considered a monocyte activation marker that links this population to hypercoagulability, persistent inflammation, and atherogenesis. The similar levels of TF^+^ monocytes between groups for all the evaluated subsets with or without stimuli indicate that the increased levels of soluble TF observed in some studies^4,39,44,47–49^ must have originated from sources other than monocytes.

Our work also showed increased levels of markers of endothelial activation, especially VCAM-1 and ICAM-1, in HICs. These proteins are cellular adhesion molecules expressed in response to endothelial activation, and their increase was associated with HIV infection in other studies^28–31,92,93^. Here, we observed similar levels for both molecules between cART and VC, in contrast to studies that observed reduced levels in response to ART^30,92,93^, although similar to a recent study that observed only a slight alteration in individuals with viremia < 3000 copies/mL submitted for treatment ^29^. Our results reinforce VCAM and ICAM as molecules as the levels are not normalized by ART. However, they indicate that ART can improve the level of other endothelial activation markers in VCs, such as AGP.

For ECs, we also observed persistent levels of VCAM-1 and ICAM-1 when compared to HIVneg, although the last was only in unadjusted data. The study of Sereti et al.^29^ observed similar results for individuals with VL < 50 copies/ml in contrast to what was observed for other markers evaluated in the study; the levels of both molecules were lower in EC than in cART, indicating that ECs could have mechanisms to control endothelial activation that are better than those achieved with ART, although not normalized.

Our study also demonstrated increased levels of ST2 in VCs compared to HIVneg. ST2 is a biomarker for cardiac stress^94^ associated with all-cause mortality in HIV-infected individuals^95,96^. Together with D-dimer, tPA, VCAM-1, ICAM-1, and AGP-2 levels observed in our study indicate that VCs could present higher susceptibility to CVD than EC and cART individuals.

The lack of a group of HIV-infected individuals who were non-HIC and non-treated should be considered a limitation for the measure of markers of CVD risk for HICs vs. untreated HIV non-controllers. This aspect could also be better addressed if the follow-up time of the study groups after sample collection was long, thereby allowing for the evaluation of CVD outcomes. Despite that, the long-term follow-up from HIV diagnosis and clinical history available for the HIV-infected individuals included in our study allowed for a better classification of HIC profiles and the evaluation of clinical stability in the years prior to sample collection. Besides that, the low number of HICs in our study, mainly VCs, could have impacted some analyses. This lack of patients is not only a consequence of the rarity of the profile but also a consequence of the universal access to ART independent of the immune status that had been institutionalized since 2013 in Brazil, making it difficult to identify new HICs and leading to the initiation of therapy in most identified VCs. In addition, a longer time since HIV diagnosis for the cART group compared to ECs could be seen as a limitation, as a longer time with detectable viremia could impact immune activation even after ART start. Despite that, we understand that a possible bias related to this factor is not accountable, as most individuals in the cART group started treatment within 3 years after diagnosis and should have unknown years of detectable viremia prior to diagnosis. In addition, the long time of antiretroviral treatment should be able to revert most of the damage caused by the time of uncontrolled viremia, attenuating the effect of long term detectable viremia in inflammation.

We verified that the level of markers associated with the risk for CVD development in ECs is lower or similar to the risk that exists for ART-suppressed individuals. Although previous studies with EC had observed signs of higher comorbidity risk^3,12,57,58^, we only observed increased platelet activation when comparing EC with cART, similarly to studies that did not identify increased risk in Ecs compared to ART-suppressed individuals^60–62^. However, the persistence of altered levels for several inflammation markers suggests that alternative therapies aiming to lower residual inflammation could benefit EC, including those under ART presenting high levels of inflammation.

In general, our data showed that VC displays increased levels of inflammation and coagulation markers associated with CVD risk. Meanwhile, EC shows signals of lower but persistent inflammation, comparable to the cART group, indicating the potential benefits of alternative therapies to decrease inflammation in this group.

## Methods

### Study population and ethical statement

Thirteen HICs with HIV-1 infection and spontaneous virological suppression for at least 5 years were recruited at the Instituto Nacional de Infectologia Evandro Chagas/Fiocruz and clinically followed annually or semi-annually. HICs were further divided into two groups: (1) ECs (n = 8) if plasmatic viral load (pVL) measures were below the lower limit of detection (< LLOD of 40–80 copies/mL, depending on the pVL test used at the time) during the follow-up; (2) VCs (n = 5) if most (≥ 70%) pVL determinations were > LLOD and < 2,000 copies/mL during the follow-up. Occasional pVL measurements above the upper limits were accepted during the follow-up of the HICs. Additionally, two groups were recruited as control groups: (1) HIV-1 infected individuals with pVL suppressed by ART for at least 2 years (cART; n = 18) matched with HICs by sex, age, hypertension, and diabetes diagnosis; (2) HIV-1-uninfected individuals (HIVneg; n = 18) matched by sex and age with HICs.

All the recruited individuals were subjected to anthropometry, the alcohol use disorders identification test (AUDIT), and a questionnaire regarding personal and family history of CVD/diabetes, physical activity frequency, and drug use/smoking habits. Medical history was available for the EC/VC/cART groups for additional information. Individuals with dyslipidemia, diabetes, and hypertension were identified as individuals under treatment to control cholesterol, glucose, and arterial pressure levels.

Ten years Framingham risk-score for CVD was measured with a calculator from the Framingham Heart Study website^100^. Both IMC and cholesterol-based calculation models were performed.

All participants provided written informed consent, and both the INI-Fiocruz and IOC-FIOCRUZ Ethical Committee Boards approved the study. All experiments were performed in accordance with relevant guidelines and regulations regarding studies with human subjects.

### Sample collection and preparation

Blood samples were collected after ≥ 8 h of fasting using heparin and EDTA vacuum tubes. The CD4^+^/CD8^+^ T lymphocyte counts and HIV-1 pVL were performed as routine clinical procedures. The remaining blood was used to isolate peripheral blood mononuclear cells (PBMCs), perform a venereal disease research laboratory (VDRL) test, and for an assessment of laboratory markers (CBC, lipid, and liver profile panel, blood glucose, and creatinine levels).

In addition, 10 mL of blood was slowly drawn in a syringe containing 15% v/v of ACD (38 mM citrate, 85 mM sodium citrate, 135 mM anhydrous dextrose, pH 5.1) pre-heated at 37 °C. ACD samples were processed within an hour after collection to prevent artificial platelet activation.

### Flow cytometry

For platelet activation analyses, ACD whole blood was diluted in Hepes-Tyrode buffer (137 mM NaCl, 2.8 mM KCI, 10 mM HEPES, 1 mM MgCl2, 12 mM NaHCO3, 0.4 mM NaH2PO4, 5.5 mM glucose, 0.35% SFB, pH 7,4), previously heated at 37 °C and incubated at room temperature. A replicate sample was simultaneously stimulated with a 160 nM concentration of phorbol 12-myristate 13-acetate (PMA) as the positive activation control. After incubation, the samples were stained with anti-CD41 APC and anti-CD62P PE antibodies. The stained samples were fixed with PBS-PFA 4% and analyzed with a FACS Calibur flow cytometer (BD Biosciences, USA).

For T cell activation and monocyte subset analyses, PBMCs were thawed, divided into two tubes, and incubated for three hours, with and without 1 µg/ml of LPS (Sigma-Aldrich, USA), as stimuli in RPMI 1640 (Sigma-Aldrich, USA) with 10% FBS (Gibco, Thermo Fisher Scientific, USA) at 37 °C, under 5% CO_2_ and controlled humidity. For T cell activation, unstimulated cells were stained with FVS450 for dead cell exclusion, anti-CD3 V500, anti-CD4 APC, anti-CD8 PE, anti-CD38 BB515, and anti-HLA-DR PERCP (all from BD Biosciences, USA). For monocyte subset analyses, both unstimulated and stimulated PBMCs were stained with anti-CD3 FITC, anti-CD14 AF700, anti-CD16 APC, and anti-TF PE. Background fluorescence for TF expression was assessed using a PE IGG1, k, isotype control. After staining, samples were fixed with PBS-PFA 1% and acquired on a BD FACSAria™ IIu flow cytometer (BD Biosciences, USA).

All analyses were performed with FlowJo™ v10.5 software (BD Life Sciences, USA)^97^. Gate strategies are described in Supplemental Figs. 5–7.

### Plasmatic inflammatory markers

Plasmatic levels of IL-6, IL-10, MCP-1, TNF-α, D-dimer, ST2, VCAM-1, and ICAM-1 were assessed using a custom ProcartaPlex multiplex immunoassay (Invitrogen, EUA) according to the manufacturer’s instructions and a MAGPIX reader (Luminex Corp, EUA). Plasmatic levels of Angiopoietin-1, Angiopoietin-2, CD40L, CD62P, PAI-1, TF, TFPI, Tpa, and vWF-A2 were assessed by ELISA with Duoset Elisa Kits (R&D Systems, EUA).

### Statistics

We performed Kruskal–Wallis ANOVA with rank tests, followed by post-hoc group pairwise comparisons with Mann–Whitney tests, with *p*-value adjustment for multiple comparisons through a Dunn’s test for the descriptive analyses of continuous numerical variables. For categorical/nominal variables, Fisher’s exact tests were used to evaluate independence among groups.

Correlation analyses were performed using the Spearman method and considered if *p* < 0.05 and rho > 0.3 in both models. Data showed in graphs and correlations were classified based on Spearman rho values as weak (rho ± 0.3–0.4), moderate (rho ± 0.4–0.7), and strong (rho ± 0.7–1).

For the inferential analyses, we fit multiple linear fixed-effects models, where the fixed systematic component of models was composed of diverse compositions of viremic controller groups and confounding variables (age, sex, and traditional risk factors for CVD: hypertension, dyslipidemia, diabetes, smoking, IMC, and total/LDL cholesterol). The results are presented graphically and in tables for the estimated mean marginal effects and their 95% confidence intervals, where all the other variables in the multiple linear models were kept at their mean values or equal proportions, and by contrast constructed from these estimated mean marginal effects. For the former, p-values were corrected by the number of comparisons with the reference level (type I comparison-wise error) by Tukey’s honest significant difference (HSD) method. Inferential analyses were performed using statistical software R version 4.1.1 (R Foundation for Statistical Computing, Austria)^98^, packages ‘lme4’, ‘emmeans’, and its dependencies. Graph construction and Mann–Whitney analyses were performed in GraphPad Prism v9 (GraphPad Software, USA, www.graphpad.com). Statistical significance was accepted as *p* < 0.05 for all analyses.

## Supplementary Information

Supplementary Information.

## Data Availability

The raw data generated and analyzed during the current study are available and can be shared by the corresponding author upon request.
