# Supplementary material for: Increased biomarkers of cardiovascular risk in HIV-1 viremic controllers and low persistent inflammation in elite controllers and art-suppressed individuals
Source: Sci Rep. 2022 Apr 21;12:6569. doi: 10.1038/s41598-022-10330-9 (PMC9023525; doi:10.1038/s41598-022-10330-9)
Supplement: Supplementary file 1 — Supplementary Information. [file 41598_2022_10330_MOESM1_ESM.docx]

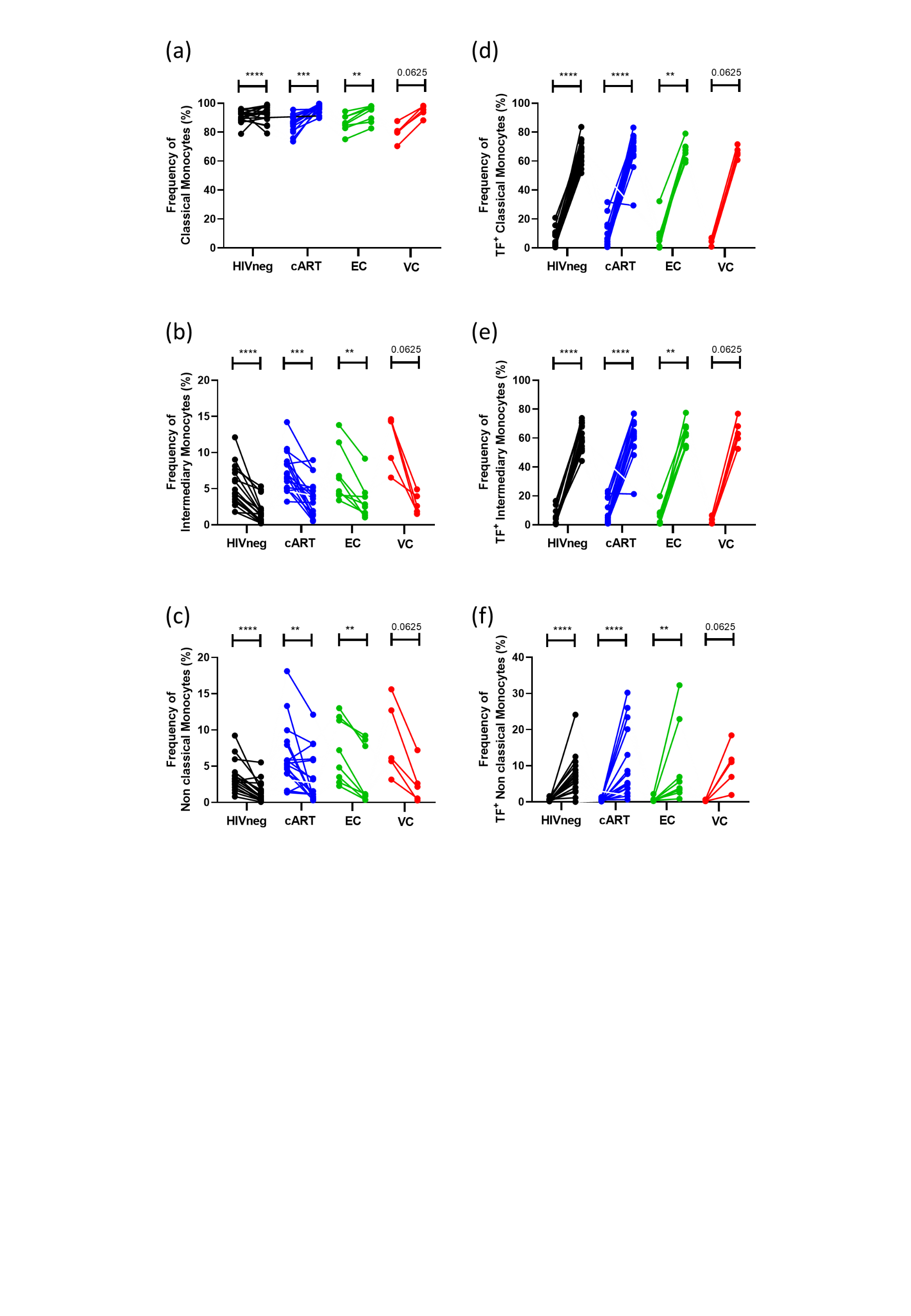


**Supplemental Figure 1. Variation in the frequency of monocytes subsets and TF expression in unstimulated and stimulated samples for the studied groups.**

(A-C) Variation between the frequency of classical (A), intermediate (B) and (C) non classical monocytes between LPS stimulated and unstimulated samples; (D-F) Variation between the frequency TF+ cells on classical (A), intermediate (B) and (C) non classical monocytes between PMA stimulated and unstimulated samples; P-values were calculated using the Wilcoxon matched-pairs signed-rank test in GraphPad Prism and are represented as follows: * p < 0.05; **p < 0,01; ***p < 0,001; ****p < 0,0001. Graphs were plotted with Graphpad Prism v9.


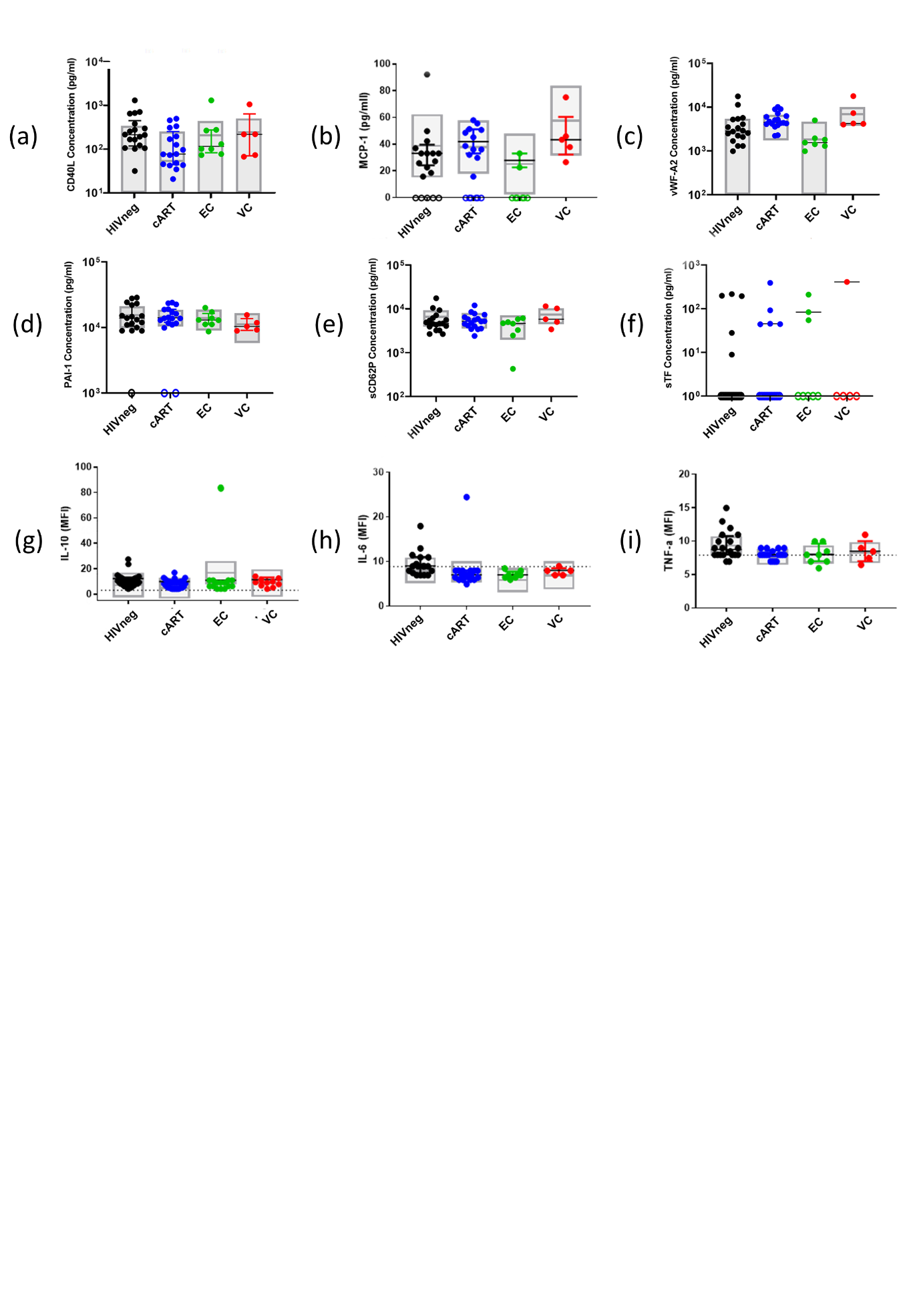


**Supplementary Figure 2. Plasma levels of inflammatory markers with similar levels in HICs and control groups**

The graphs represent the concentrations measured by multiplex Luminex or ELISA assay of: (a) CD40L; (b) MCP-1; (c) vWF-A2; (d) PAI-1; (e) sCD62P; (f) sTF; (g) IL-10; (h) IL-6; (i) TNF-a; For graphs g-i, Luminex MFI values are shown instead of concentration and the dashed line indicates the background fluorescence threshold. Open circles on the X axis represent samples with undetectable levels of the marker;

Colored horizontal bars represent the IQR and sample median, while gray boxplots represent linear model estimated adjusted means and 95% confidence intervals (CI 95%). Comparisons of means among groups were performed by contrasts/differences obtained after both bi- and multivariate-linear models fitted by ordinary least square regressions. P-values were corrected by the Tukey Honest Significant Difference post hoc method and represented as: * p < 0.05; **p < 0.01; ***p < 0.001; ****p < 0.0001. Graphs were plotted with Graphpad Prism v9.

**
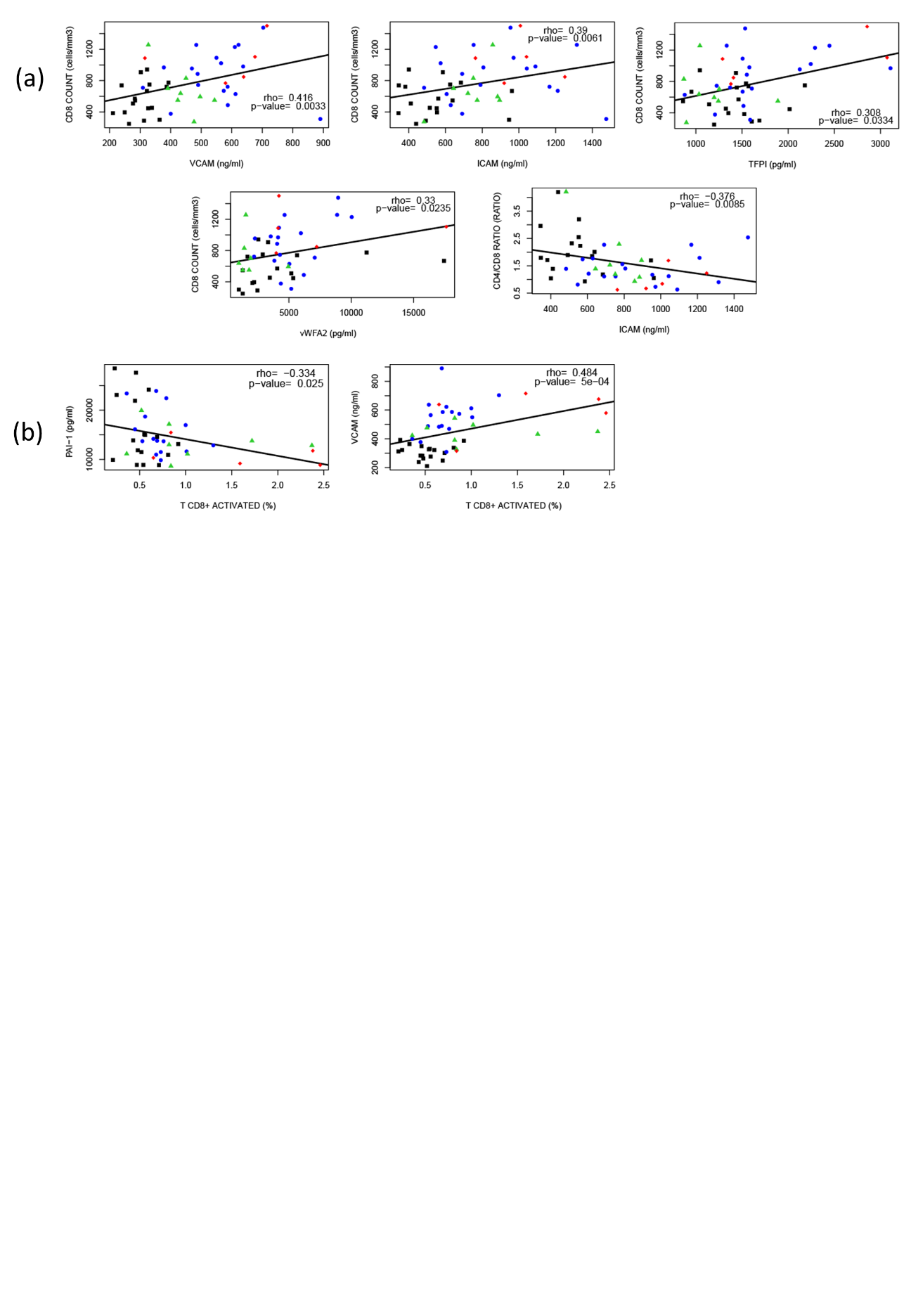
**

**Supplementary Figure 3. Correlations between T cells counts, T cell activation, and plasma inflammatory markers.**

(a) Significant Spearman correlations between inflammatory markers evaluated in the study and T CD8 count; (b) Significant Spearman correlations between inflammatory markers evaluated in the study and CD8^+^ and CD4^+^ activated T cells. Correlation graphs were plotted with R software v4.1.

**
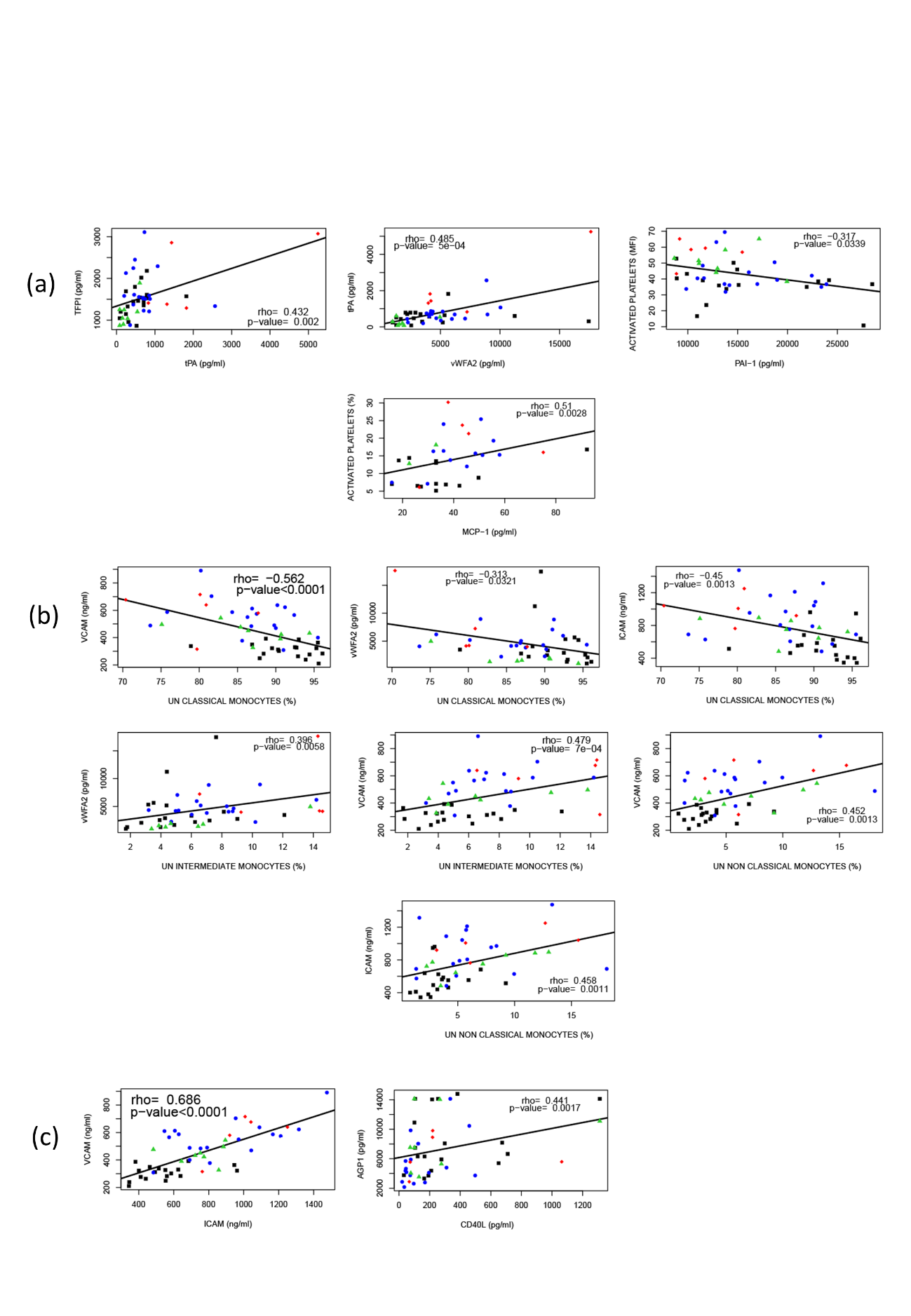
**

**Supplementary Figure 4.** **Correlations between platelet activation, monocyte subsets, and endothelium activation and plasma inflammatory markers.**

(a) Significant Spearman correlations between inflammatory markers evaluated in the study and the frequency or MFI of activated platelets; (b) Significant Spearman correlations between inflammatory markers evaluated in the study and the frequency of classical, intermediate, and non-classical monocytes; (c) Significant Spearman correlations between inflammatory markers evaluated in the study and the concentration of endothelium activation markers. Correlation graphs were plotted with R software v4.1.

**
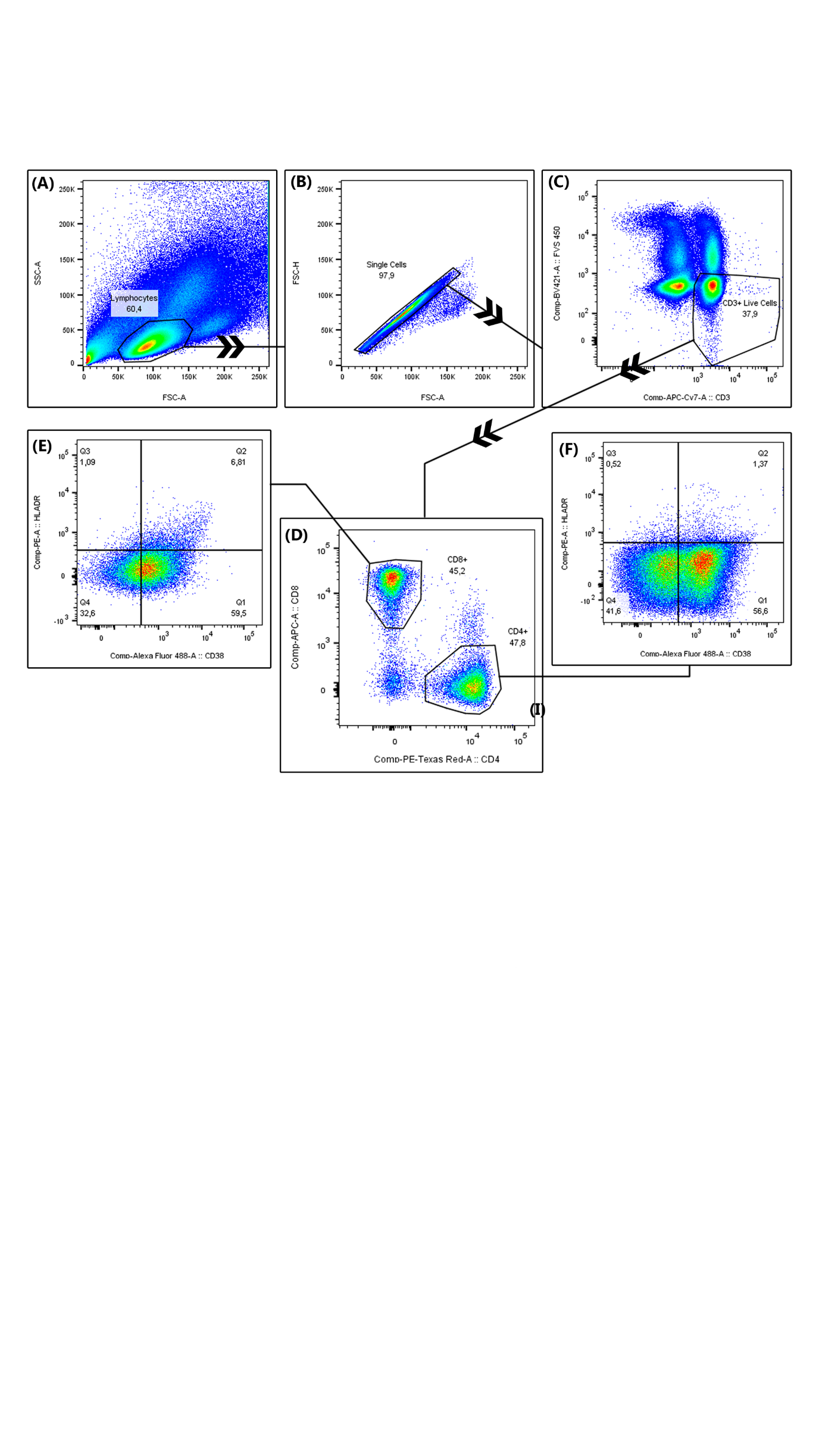
**

**Supplemental Figure 5. Gate Strategy for evaluation of T-cell Activation by flow cytometry.**

Lymphocytes were characterized by the patterns of forward and side scatter, followed by selection of FVS-CD3^+^ cells. Activated T cells were characterized as CD38+HLA-DR+ T-cells in CD4^+^ and CD8^+^ subsets. Arrows represent the flow of analysis. Dotplots were obtained with FlowJo™ v10.5.

**Supplemental Figure 6. Gate Strategy for evaluation of Platelet Activation by flow cytometry.**

Total platelets were identified based on forward and side scatter patterns, followed by selection of CD41+ cells. PMA stimulated samples were compared with unstimulated samples to identify the limits between CD62P^+^ and CD62P- populations. Arrows represent the flow of analysis. Dotplots were obtained with FlowJo™ v10.5.

**
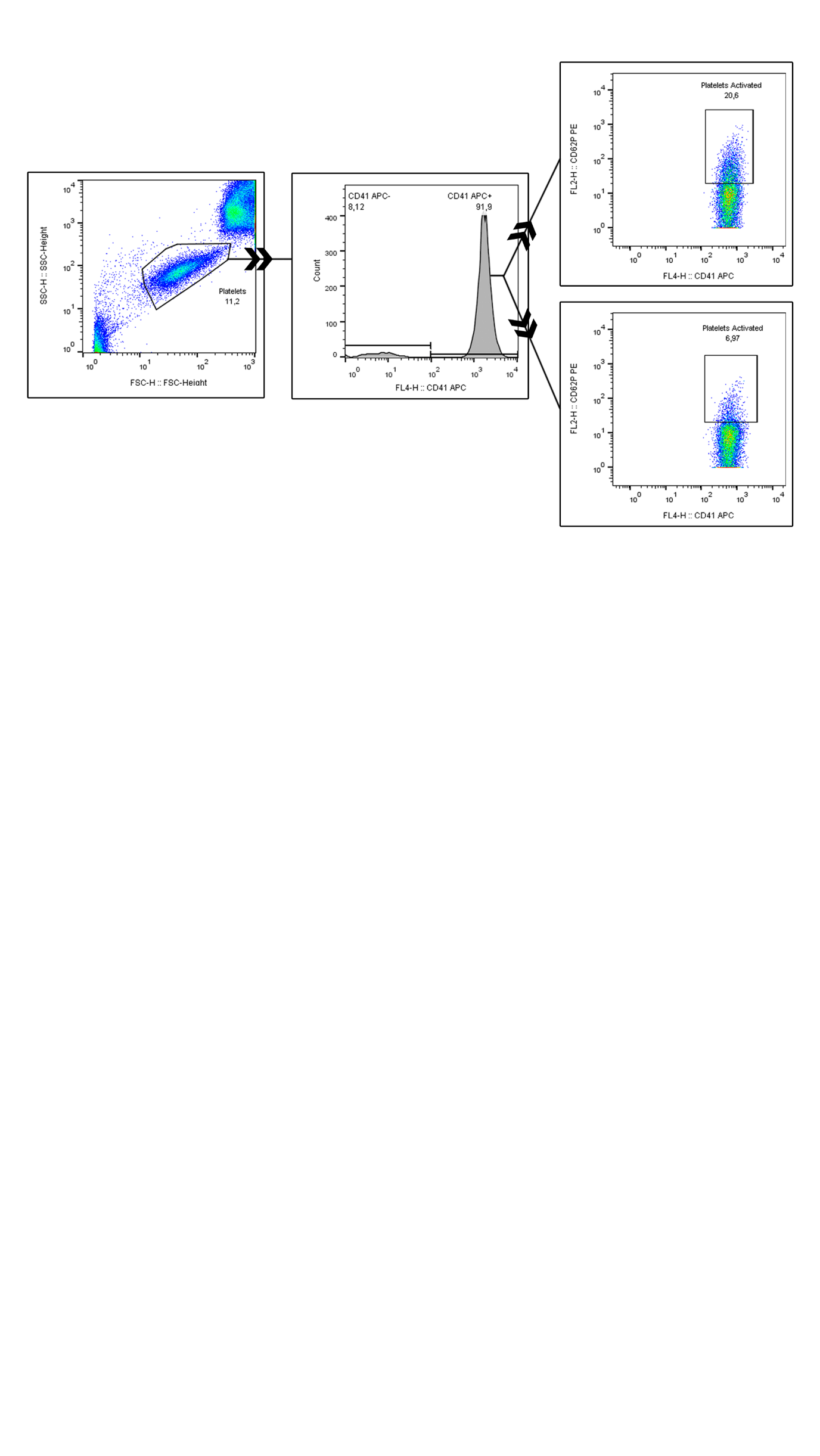
**

**
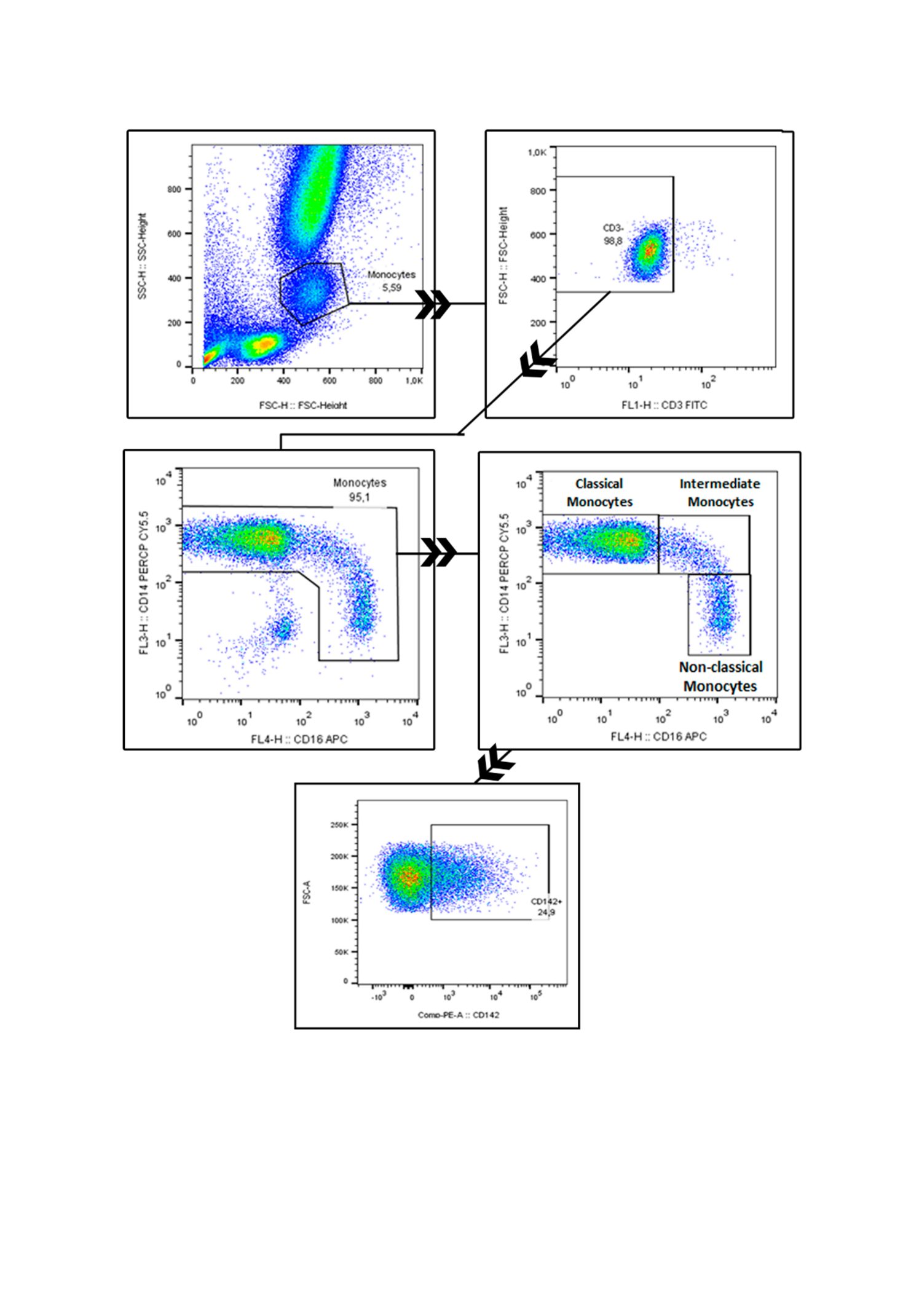
**

**Supplemental Figure 7. Gate Strategy for evaluation of monocyte subsets by flow cytometry.**

Total Monocytes were identified based on forward and side scatter patterns, followed by selection of CD3- cells. MPAs were characterized as CD14^+^CD41^+^ cells in the total monocytes population. For subsets, an exclusion gate for CD14^-^CD16^-^ were used to exclude non-monocytes and the subsets of classical (CD14^++^CD16^-^), intermediate (CD14^++^CD16^+^) and non classical monocytes (CD14^+^CD16^++^) were defined based on diferential expression of CD14 and CD16. Further, Tissue fator (CD142) expression was evaluated within the whole monocytes population and each subset. Arrows represent the flow of analysis. Dotplots were obtained with FlowJo™ v10.5.

| **Supplemental Table 1.** Therapeutic schemes and the respective drugs used by ART-treated individuals from the study | | |  |
| --- | --- | --- | --- |
| **INDIVIDUAL** | **THERAPEUTIC SCHEME** | **DRUGS** |  |
| cART01 | 2NRTI+1NNRTI | AZT/3TC/EFV |  |
| cART02 | 2NRTI+ 1NNRTI | AZT/3TC/EFV |  |
| cART03 | 1NRTI+1INI+bPI | 3TC/DGV/DRV/RTV |  |
| cART04 | INI+bPI | DTG/DRV/RTV |  |
| cART05 | 2NRTI+1INI | TDF/3TC/DTG |  |
| cART06 | 2NRTI+1NNRTI | TDF/3TC/EFV |  |
| cART07 | 2NRTI+1NNRTI | TDF/3TC/EFV |  |
| cART08 | 2NRTI+1NNRTI | TDF/3TC/EFV |  |
| cART09 | 2NRTI+bPI | TDF/3TC/LPV/RTV |  |
| cART10 | 2NRTI+1NNRTI | TDF/3TC/EFV |  |
| cART11 | 2NRTI+1NNRTI | TDF/3TC/EFV |  |
| cART12 | 2NRTI+bPI | TDF/3TC/ATV/RTV |  |
| cART13 | 2NRTI+bPI | TDF/3TC/DRV/RTV |  |
| cART14 | 2NRTI+bPI | ABC/3TC/DRV/RTV |  |
| cART15 | 2NRTI+1PI | TDF/3TC/DRV |  |
| cART16 | 2NRTI+1NNRTI | TDF/3TC/NPV |  |
| cART17 | 2NRTI+1INI | TDF/3TC/DTG |  |
| cART18 | 2NRTI+1INI | TDF/3TC/DTG |  |
| **NRTI: Nucleoside reverse transcriptase inhibitor; NNRTI: Non-nucleoside reverse transcriptase inhibitor; bPI: Boosted Protease Inhibitor; INI: Integrase Inhibitor; AZT: Zidovudine; 3TC: Lamivudine; EFV: Efavirenz; DGV: Dolutegravir; DRV: Darunavir; RTV: Ritonavir; LPV: Lopinavir; ATV: Atazanavir; NPV: Nevirapine** | | |  |
|  |  |  |  |
|  |  |  |  |
|  |  |  |  |
